# Perioperative Pain Relief in Pediatric Tonsillectomy: Current Strategies and Future Directions

**DOI:** 10.3390/children12121619

**Published:** 2025-11-27

**Authors:** Harshini Medikondu, Annie Chen, Doreen Soliman, Samir Yellapragada, Senthilkumar Sadhasivam, Mihaela Visoiu

**Affiliations:** Department of Anesthesiology and Perioperative Medicine, University of Pittsburgh School of Medicine, UPMC Children’s Hospital of Pittsburgh, Pittsburgh, PA 15224, USA; medikonduh@upmc.edu (H.M.); chen.annie@medstudent.pitt.edu (A.C.); solimande@upmc.edu (D.S.);

**Keywords:** pediatric tonsillectomy, postoperative pain, multimodal, analgesia, opioid

## Abstract

**Highlights:**

**What are the main findings?**

- Post-tonsillectomy pain in children arises from multiple mechanisms including surgical trauma, inflammation, and secondary wound healing, and follows a characteristic biphasic course.
- The most effective management strategy is a multimodal analgesic regimen combining acetaminophen, NSAIDs, and perioperative dexamethasone, supported by local anesthetic techniques and complementary home-based measures.
- Careful selection and timing of analgesics significantly reduce pain intensity, limit opioid use, and improve early oral intake and recovery.

**What is the implication of the main finding?**

- Implementing an individualized multimodal approach promotes safer opioid-sparing pain control and enhances recovery after pediatric tonsillectomy.
- Understanding pain mechanisms and pharmacologic interactions enables clinicians to tailor treatment, prevent complications, and reduce hospital readmissions.

**Abstract:**

Postoperative pain following pediatric tonsillectomy remains a significant clinical challenge. Inadequate management leads to delayed recovery, dehydration, and increased healthcare utilization. Multimodal analgesia, including combining systemic analgesics, local anesthetic techniques, and adjunctive medications, has emerged as the standard of care. This review highlights current perioperative pain management strategies, including recent evidence on multimodal and opioid-sparing approaches. Local infiltration and nerve blocks provide site-specific pain relief, reducing opioid requirements and improving patient comfort. A comprehensive understanding and careful selection of analgesic regimens are crucial for enhancing outcomes in this population.

## 1. Introduction

Tonsillectomy is one of the most commonly performed surgical procedures in pediatric patients [1,2]. Postoperative pain following pediatric tonsillectomy is typically moderate to severe, often prolonged and peaking in the first 24–48 h with a second peak around days 5–8 related to eschar sloughing.

Prospective cohort data and recent reviews consistently report pain intensity in the moderate-to-severe range for many children, underscoring the need for reliably scheduled, multimodal analgesia [3,4,5]. Effective perioperative pain control is crucial for facilitating oral intake, reducing the risk of dehydration, and minimizing the likelihood of hospital readmissions [4]. This review synthesizes current evidence on the severity and temporal pattern of postoperative pain after pediatric tonsillectomy, examines published intervention trials (pharmacologic and regional), and provides evidence-based recommendations for perioperative analgesic practice.

Post-tonsillectomy pain is primarily nociceptive, arising from exposed pharyngeal muscle and local inflammation, with neuropathic features contributing in some patients. Clinically, pain follows a biphasic course, peaking early (24 to 48 h) and often again around days 5 to 8 as the fibrin eschar separates, which supports the need for sustained and scheduled multimodal analgesia throughout the first postoperative week [3,4,5].

Clinical studies identify several predictors of more severe or prolonged post-tonsillectomy pain: older pediatric age (school aged children/adolescents), obstructive sleep apnea (OSA), high preoperative anxiety, high baseline pain sensitivity, and prior painful medical experiences. These patients more frequently require intensified perioperative planning (preoperative counseling, scheduled multimodal regimens, and closer post discharge follow up) [4,5,6,7]. Historically, opioids were the mainstay of analgesia; however, concerns regarding respiratory depression and other adverse effects have shifted practice toward multimodal approaches. These strategies integrate systemic analgesics, regional anesthetic techniques, and adjunctive agents to optimize pain relief while minimizing opioid exposure [8].

The healing process contributes to a characteristic biphasic pain pattern. The tonsillar fossa heals via secondary intention, forming a protective fibrin eschar that increases in size over the first 3–4 postoperative days. This eschar begins to shed around day 7, exposing underlying nerve fibers and damaged muscle to mechanical stimuli from swallowing, which can exacerbate pain and delay remucosalization, often completed by the end of the second week [9]. This process explains why pain often peaks several days after surgery and may persist for up to two weeks.

Furthermore, pain can be compounded by factors related to surgical positioning. The placement of the Boyle–Davis mouth gag can cause pressure-induced ischemia and venous congestion of the tongue, leading to postoperative pain and swelling, as well as stretching of the temporomandibular joint [10]. Patients also frequently experience referred otalgia, as pain signals from the tonsillar bed (innervated by the glossopharyngeal nerve) converge on the same spinal nucleus (nucleus solitarius) as sensory fibers from the ear [11].

Inadequate pain control after pediatric tonsillectomy triggers a detrimental cycle of decreased oral intake, leading to dehydration and a heightened risk of hospital readmission. This increases the potential for postoperative hemorrhage due to pain-induced agitation and hypertension. This state of dehydration can dangerously concentrate renally cleared analgesics like non-steroidal anti-inflammatory drugs (NSAIDs) and acetaminophen, elevating the risk of acute kidney injury and hepatotoxicity. Evidence indicates that nonsteroidal anti-inflammatory drugs can precipitate acute kidney injury in children, particularly in the setting of dehydration or reduced renal perfusion, even when administered at therapeutic doses [12,13,14,15,16]. Similarly, acetaminophen hepatotoxicity may occur following overdose or repeated supratherapeutic dosing, especially during illness, fasting, or dehydration, due to accumulation of the reactive metabolite N-acetyl-p-benzoquinoneimine [17,18,19,20,21].

Furthermore, poorly managed acute pain can induce central sensitization, raising the long-term risk of chronic pain conditions, and contribute to significant psychological sequelae such as medical trauma, needle phobia, and heightened anxiety for both the child and parents [7]. Similarly, the efficacy and safety of opioids like codeine and tramadol are highly dependent on pharmacogenetics. These drugs are prodrugs that require metabolic activation by the cytochrome P450 enzyme CYP2D6. Patients with genetic polymorphisms leading to ultra-rapid metabolizer phenotypes are at risk for toxic levels of active drug and life-threatening respiratory depression, which is why they are now contraindicated in children following tonsillectomy [22]. A comprehensive understanding of these mechanisms and risks is therefore critical for selecting effective and safe analgesic regimens.

## 2. Multimodal Analgesia

### 2.1. Acetaminophen

Acetaminophen is a cornerstone of multi-modal analgesia for pediatric tonsillectomy, providing effective pain relief while directly mitigating opioid requirements. Acetaminophen primarily exerts its effects through central inhibition of cyclooxygenase (COX) enzymes and modulation of descending serotonergic pathways, thereby providing foundational analgesia [23].

This foundational role refers to acetaminophen’s ability to act as the base layer of multimodal analgesia, upon which other agents can build additive or synergistic effects [24]. This reduction in baseline pain sensitivity allows adjunct medications such as nonsteroidal anti-inflammatory drugs, local anesthetics, or opioids used for breakthrough pain to act more efficiently and at lower doses [25]. In pediatric tonsillectomy, this foundational effect contributes to smoother pain trajectories, fewer opioid requirements, and better tolerance of oral intake, which together form the primary goals of modern opioid-sparing perioperative care [1]. Acetaminophen’s analgesic effect is further enhanced when combined with nonsteroidal anti-inflammatory drugs (NSAIDs), forming a well-established synergistic core of multimodal analgesia [24,26].

Consequently, this evidence-based combination is a pivotal strategy for developing effective, opioid-sparing analgesic regimens. The efficacy of acetaminophen is maximized through scheduled, around-the-clock administration. Clinical practice guidelines strongly endorse this strategy to maintain consistent therapeutic plasma levels and provide superior pain control compared to “as-needed” dosing [25]. The availability of an intravenous formulation is particularly valuable in the immediate postoperative period, ensuring reliable analgesia when oral intake is not feasible. Evidence demonstrates that intravenous acetaminophen effectively reduces both early and delayed pain. It significantly decreases the need for rescue morphine in the post-anesthesia care unit, enhancing early recovery safety [27].

The strategic role of acetaminophen extends beyond the hospital, forming the bedrock of successful outpatient opioid-sparing protocols. When integrated into a standardized pain management regimen, scheduled acetaminophen (often combined with ibuprofen) provides sufficient analgesia for many patients, drastically reducing or even eliminating the need for opioids at discharge. This approach directly addresses critical concerns regarding opioid-related adverse effects, such as nausea, vomiting, and respiratory depression, thereby promoting safer recovery at home. Clinical studies have proven that such protocols, with acetaminophen as a core component, are highly effective in managing pain while minimizing overall opioid exposure, facilitating a safer and more comfortable recovery for pediatric patients [2].

### 2.2. Non-Steroidal Anti-Inflammatory Drugs (NSAIDs) in Pediatric Tonsillectomy

As noted above, scheduled acetaminophen combined with NSAIDs such as ibuprofen provides synergistic analgesia and forms the foundation of multimodal perioperative pain control (Table 1). 

NSAIDs primarily inhibit peripheral cyclooxygenase (COX) enzymes, reducing prostaglandin-mediated inflammation and complementing the central action of acetaminophen [23,26]. The AAO-HNS 2019 Clinical Practice Guideline [1] recommends combining these analgesics while minimizing opioid use, particularly in children under 12 years and multiple clinical trials have confirmed that the acetaminophen-NSAID combination reduces postoperative pain scores and opioid requirements more effectively than either drug alone [1,23,26,28,29]. The PROSPECT protocol (2021) echoes this approach, recommending intraoperative dexamethasone and reserving opioids solely for rescue analgesia [30].

**Table 1:** NSAIDs for Pediatric Tonsillectomy Pain Management.

Drug	Typical Pediatric Dose	Analgesic Benefit	Bleeding Risk Evidence	Notes/Guidelines
Ibuprofen	5–10 mg/kg q6–8 h (max 40 mg/kg/day)	Adequate analgesia reduces opioid rescue use	No significant increase in PTH [31]	AAO-HNS 2019: Strongly recommended
Ketorolac	0.5 mg/kg IV single intraoperative dose	Opioid-sparing in some trials	Mixed evidence; slightly higher bleeding signal in adults; pediatric data are less clear	Use cautiously; not first-line
Diclofenac	1 mg/kg PO/PR q8 h (max 75 mg/day)	Some pediatric data for analgesic benefit	Limited tonsillectomy data; no clear bleeding signal	More common in Europe; less used in the US
Ketoprofen	0.5–1 mg/kg IV at induction, then 3 mg/kg over 24 h; OR 3–5 mg/kg/day PO divided q6–8 h	Adequate analgesia reduces opioid requirement	Limited pediatric tonsillectomy data; generally, no increased bleeding in controlled studies	Use with caution; monitor for GI or renal side effects.
Celecoxib	3 mg/kg PO q12 h (max 400 mg/day)	Reduces early postoperative pain and opioid use with a favorable safety profile	No increased risk of post-tonsillectomy hemorrhage requiring surgery	Effective COX-2–selective option with favorable safety profile; further prospective studies recommended before routine use

Historically, concerns about NSAID-associated bleeding have limited their use. A 2022 meta-regression study by Losorelli et al. examined ibuprofen at doses of 5 mg/kg and 10 mg/kg and found no statistically significant increase in post-tonsillectomy hemorrhage [31]. Similarly, a 2013 Cochrane review of 15 trials enrolling both adult and pediatric patients found no statistically significant evidence that NSAIDs increase the risk of perioperative bleeding requiring surgical intervention, reoperation, or readmission. However, the analysis could not exclude a potential clinically substantial increase in the risk of reoperation due to wide confidence intervals [32].

In contrast, a 2024 analysis of ketorolac in 17,000 matched pediatric tonsillectomy cases revealed increased risk of both primary hemorrhage (OR 2.16, 95% CI 1.35–3.44) and secondary hemorrhage (OR 1.37, 95% CI 1.06–1.79) when administered on the day of surgery [33]. The association between ketorolac and post-tonsillectomy hemorrhage appears to be age-specific. A landmark study found that while ketorolac significantly increased the risk of hemorrhage in adults, it did not do so in children [34]. More recent studies have supported this finding of relative safety in the pediatric population [9,34].

A systematic review and meta-analysis published in Scientific Reports in January 2025 analyzed various pain management strategies after tonsillectomy. The study found that preoperative or intraoperative administration of NSAIDs, including ketoprofen, significantly reduced postoperative pain intensity compared to placebo or no treatment. This reduction was consistent across multiple time points, including the first 24 h, and on days 1, 3, and 7 postoperatively [35].

Studies by Salonen and Kokki have demonstrated the efficacy of rectal ketoprofen for post-tonsillectomy analgesia in school-aged children (mean age ~7 years). Their research found that a dose of 2 mg/kg was associated with a significant reduction in opioid consumption and improved pain scores compared to placebo. However, the rectal route may present practical challenges related to patient acceptance and adherence outside of a controlled research setting, limiting its widespread use in favor of oral or intravenous alternatives [36,37].

Selective COX-2 inhibitors have also been explored as alternatives to traditional NSAIDs in pediatric tonsillectomy. In a randomized controlled trial, Murto et al. demonstrated that celecoxib significantly reduced early postoperative pain and opioid consumption compared to placebo, with no increase in adverse events; pharmacogenetic variability was also noted, with children carrying the CYP2C9*3 allele showing enhanced analgesic effects [38]. Building on this, So et al. conducted a large retrospective cohort study of over 6000 pediatric tonsillectomy patients and found that celecoxib use was not associated with an increased risk of post-tonsillectomy hemorrhage requiring surgery [39]. These findings suggest celecoxib may provide effective analgesia while maintaining an acceptable safety profile, though further prospective data are warranted before routine use.

### 2.3. Role of Opioids

The role of opioids in pediatric tonsillectomy analgesia has been dramatically redefined, shifting from a first-line treatment to a limited, rescue-only therapy due to significant safety concerns. This paradigm shift is driven by evidence that routine opioid use, particularly codeine and hydrocodone, poses a substantial risk of fatal respiratory depression in children, leading to FDA black box warnings against their use in this population [1,40]. Consequently, multi-modal, opioid-sparing protocols built on scheduled acetaminophen and NSAIDs like ibuprofen are now the standard of care, as they provide adequate analgesia without increasing bleeding risk and have been proven to significantly reduce or even eliminate the need for opioids in most patients [2,41]. When severe breakthrough pain occurs, low-dose, weight-based oxycodone is often preferred over other opioids for rescue therapy. Still, its use must be carefully weighed against the risk of adverse effects, which include nausea, vomiting, and respiratory compromise [22,42]. National initiatives and institutional quality improvement projects have successfully demonstrated that standardizing pain management protocols to minimize opioid prescribing is both safe and effective, drastically reducing opioid exposure while maintaining satisfactory pain control [41,43].

Methadone is a long-acting µ-opioid receptor agonist with additional N-methyl-D-aspartate (NMDA) receptor antagonism, producing prolonged postoperative analgesia through dual mechanisms that reduce both nociceptive and central sensitization pathways. Randomized controlled trials in pediatric tonsillectomy have evaluated intravenous methadone at doses of 0.10 to 0.15 mg per kg, demonstrating effective opioid-sparing and improved early postoperative comfort compared with short-acting opioid regimens [44]. Its intraoperative use may be considered in selected patients, particularly those with high opioid requirements or contraindications to short-acting opioids, with careful monitoring of respiratory and cardiac function to minimize adverse effects [43,44]. However, methadone should not be considered a first line outpatient opioid in children because of its long and variable half-life, risk of accumulation, and potential for delayed respiratory depression. In addition, methadone can prolong the QT interval, predisposing susceptible patients to torsades de pointes; therefore, careful patient selection, review of baseline electrocardiogram, and monitoring for QT prolongation are recommended [43]. When used appropriately, methadone is best regarded as an intraoperative adjunct for sustained analgesia within an opioid-sparing multimodal regimen, rather than a routine postoperative analgesic [43,44].

### 2.4. Pre-Emptive Analgesia

Pre-emptive analgesia refers to administering analgesics before the surgical stimulus to attenuate central sensitization, decreasing postoperative pain, and reducing opioid requirements. In pediatric tonsillectomy, this approach complements multimodal analgesia strategies and may optimize recovery. The choice of primary anesthetic agent itself can be considered a foundational pre-emptive strategy. Although prior studies have hypothesized that propofol-based anesthesia might attenuate postoperative hyperalgesia compared with volatile agents, the current evidence indicates comparable clinical efficacy between propofol and sevoflurane in terms of pain control and recovery quality [45]. Randomized controlled trials support the use of intravenous ibuprofen (10 mg/kg administered ~15 min before incision) to reduce postoperative opioid rescue requirements without increasing the risk of nausea, vomiting, or postoperative hemorrhage. However, the practical implementation of this specific protocol may be limited by operating room logistics, the higher cost of intravenous formulation, and the widespread efficacy of pre-operatively administered oral ibuprofen [46]. Similarly, the structured use of acetaminophen in combination with NSAIDs aligns with evidence-based multimodal regimens endorsed by the AAO-HNS Clinical Practice Guideline (2019)and the PROSPECT recommendations (2021) [1,30].

Dexamethasone, a potent long-acting corticosteroid, is a cornerstone of multimodal analgesia and antiemetic prophylaxis in pediatric tonsillectomy. Its mechanism of action is primarily rooted in its potent anti-inflammatory and immunosuppressive effects. It inhibits phospholipase A2, preventing the release of arachidonic acid from cell membranes and thereby suppressing the entire cascade of inflammatory mediators, including prostaglandins and leukotrienes. This reduction in local inflammation and edema at the surgical site directly correlates with reduced pain scores and facilitates earlier oral intake. Furthermore, its antiemetic effect is mediated by central inhibition of serotonin release in the chemoreceptor trigger zone (CTZ) [47,48,49].

When administered intraoperatively (0.5 mg/kg, maximum 8–20 mg), dexamethasone provides consistent benefits, including reduced pain scores, a delayed time to the first request for rescue analgesia, earlier resumption of oral intake, and a significant reduction in postoperative nausea and vomiting (PONV) [48,49]. A 2025 prospective, randomized controlled non-inferiority trial by Brooks Peterson et al. demonstrated that oral dexamethasone (0.5 mg/kg) is not inferior to the intravenous formulation in reducing postoperative nausea and vomiting and improving pain outcomes in children undergoing tonsillectomy, providing a compelling and practical alternative for perioperative care [50]. Recent evidence suggests that a short oral course of corticosteroids after tonsillectomy may provide additional analgesic benefit once patients are discharged. Shayan et al. demonstrated that oral prednisolone significantly reduced pain scores and analgesic requirements following tonsillectomy with sutures compared with standard care. This finding supports consideration of a limited, closely monitored home taper of oral corticosteroids, such as prednisolone, as part of multimodal pain management when clinically appropriate [51].

Adjunctive agents such as gabapentinoids have been explored for their potential to modulate neuropathic and central sensitization components of post-tonillectomy pain [52]. Their proposed mechanism of action involves binding to the α2δ-1 subunit of presynaptic voltage-gated calcium channels in the central nervous system. This binding inhibits the release of excitatory neurotransmitters (e.g., glutamate, substance P) involved in pain signaling, thereby dampening hyperexcitability and potentially preventing the wind-up phenomenon.

Despite this rational pharmacological basis, pediatric evidence for their efficacy specifically in tonsillectomy remains limited. A small pilot trial of gabapentin (10 mg/kg orally) suggested reduced analgesic consumption post-discharge but no significant improvement in pain scores, highlighting the need for larger, definitive studies before routine adoption can be recommended [53].

## 3. Home Management and Non-Pharmacologic Adjuncts

Pain control after pediatric tonsillectomy extends beyond medications. Effective home management, caregiver education, and simple comfort measures can substantially reduce postoperative distress, improve adherence, and promote faster recovery.

### 3.1. Structured Discharge and Caregiver Education

A critical safety principle is the absolute contraindication of codeine. This is due to its potential for unpredictable, life-threatening respiratory depression, mechanistically driven by its metabolism via the cytochrome P450 enzyme CYP2D6. Children with ultrarapid metabolizer phenotypes convert codeine to morphine too efficiently, resulting in toxic blood levels that severely depress the brainstem’s respiratory drive [2,54,55]. Consequently, the SAMBA statement strongly supports scheduled acetaminophen and ibuprofen while discouraging opioids such as codeine or tramadol, particularly in children with obstructive sleep apnea (OSA), who are at elevated baseline risk [56].

Structured discharge protocols should include (Table 2):

- Written dosing charts for scheduled analgesics
- Hydration and diet guidance (soft foods, frequent fluid intake) [1]
- Education for caregivers regarding referred otalgia (ear pain), a common and benign finding that results from shared glossopharyngeal innervation between the tonsillar fossa and ear canal. Anticipatory guidance about this symptom, which peaks around days 3–7, helps reduce anxiety and unnecessary consultations [2,11]

Caregiver counseling should cover:

- Safe alternating of acetaminophen and NSAIDs, daily maximum doses, and recognition of inadequate pain control [1,30]
- Adequate hydration and nutrition to prevent dehydration [1]
- Warning signs for secondary hemorrhage (days 5–10), dehydration, or respiratory distress [1,33]
- Non-pharmacologic strategies such as distraction, music therapy, relaxation, and comfort measures [2]
- Clear instructions for emergency contact with healthcare providers [1]

Structured caregiver education improves adherence to pain regimens, reduces complications, and enhances overall recovery outcomes.

### 3.2. Hydration, Diet, and Cryotherapy

Non-medication measures can complement pharmacologic pain relief. Ice cream and other cold foods such as popsicles or ice chips provide local cooling that decreases inflammation in the oropharynx and produces transient numbness at the surgical site [57]. This effect likely arises from vasoconstriction and desensitization of peripheral nerve endings [58,59]. In a randomized controlled trial, ice-lollies significantly lowered pain scores during the first postoperative hour compared with no intervention [60]. The American Academy of Otolaryngology recommends cold application, including the use of ice cream, as an adjunct to multimodal analgesia for pediatric tonsillectomy [1].

Beyond physical relief, these comfort measures also provide psychological reassurance to children and caregivers, fostering a sense of nurturing and emotional support even when objective pain reduction is modest.

### 3.3. Complementary Therapies

Honey has long been used as a natural soothing agent due to its anti-inflammatory and antimicrobial properties. Its role in pediatric tonsillectomy has been explored as a non-pharmacologic adjunct aimed at improving swallowing comfort and reducing analgesic needs.

In a randomized, double-blind, placebo-controlled study, Boroumand et al. (2013) found that children receiving honey plus acetaminophen had significantly lower visual-analogue-scale pain scores and reduced analgesic consumption during the first three postoperative days compared with acetaminophen alone, though benefits were short-lived [61].

A systematic review and meta-analysis by Lal et al., including eight RCTs, also suggested reduced pain, nocturnal awakenings, and analgesic use, but the overall evidence quality was rated low to very low because of small sample sizes and heterogeneous outcomes [62].

Later, Ozturk et al. highlighted honey’s broad wound-healing properties, yet evidence specific to tonsillectomy remained inconsistent [63].

Most recently, the multicenter BEE PAIN FREE Trial by Sommerfield et al. provided high-quality evidence showing that postoperative honey administration did not significantly reduce pain or opioid use compared with placebo [64].

In conclusion, although honey is safe, well-tolerated, and widely accepted by children and parents, current high-level evidence does not support its routine use as an effective analgesic for pediatric tonsillectomy. It may still serve as a comforting adjunct with psychological rather than pharmacologic benefit [61,62,63,64].

## 4. Local Infiltration and Nerve Blocks

Several local anesthetic techniques are effective components of a multimodal strategy for reducing postoperative pain and analgesic requirements in children following tonsillectomy. Bupivacaine instillation has been shown to provide superior postoperative analgesia and facilitate earlier recovery [65]. Furthermore, Ju et al. reported that combining dexamethasone with 0.2% ropivacaine for local infiltration significantly reduced postoperative pain (4–24 h), analgesic use, PONV incidence, and time to discharge, while improving oral intake, compared to ropivacaine alone [66]. This supports earlier findings that pre-incisional infiltration of local anesthetic can significantly reduce post-tonsillectomy pain in pediatric patients [67].

The glossopharyngeal nerve block has also emerged as a valuable technique for enhancing postoperative pain control. A randomized controlled trial demonstrated that its application significantly delays the time to the first request for rescue analgesia and reduces total postoperative opioid consumption [68]. Collectively, when integrated with systemic analgesics, these regional techniques form a comprehensive multimodal approach that enhances analgesic efficacy and reduces opioid exposure [30].

A recent randomized trial by Lin et al demonstrated that suprazygomatic maxillary nerve blocks (SZMB) with ropivacaine significantly reduced total opioid consumption and lowered pain scores on PACU arrival in children undergoing adenotonsillectomy. The intervention group also experienced a prolonged time to first request for rescue analgesia compared to the sham block control group. The authors concluded that SZMB is a safe and effective technique for enhancing multimodal, opioid-sparing pain management in this patient population [69].

A 2024 randomized trial by Atef et al. directly compared ultrasound-guided versus landmark-based techniques for this block in pediatric tonsillectomy. The study demonstrated that ultrasound guidance provided superior postoperative analgesia, resulting in significantly lower pain scores and reduced rescue analgesic requirements in the first 24 h compared to the blind technique, without an increase in complications [70].

## 5. Surgical Techniques for Tonsillectomy and Adenoidectomy and Their Impact on Postoperative Pain

### 5.1. Tonsillectomy Techniques

#### 5.1.1. Extracapsular (Total/Traditional) Tonsillectomy

∘ Description: Complete removal of the tonsil along with the capsule, fully exposing the pharyngeal muscles. Hemostasis is achieved via ligature, cautery, or electrocautery.
∘ Pain Implications: Exposure of pharyngeal muscles and nerve endings leads to higher postoperative pain, increased analgesic requirements, and delayed return to normal diet and activities.
∘ Pain Management Considerations: Requires scheduled NSAIDs and acetaminophen; opioids may be used for breakthrough pain [71,72].

#### 5.1.2. Intracapsular (Partial/Subtotal) Tonsillectomy

∘ Description: Tonsillar tissue is removed while leaving a thin rim of tonsillar capsule intact, minimizing exposure of pharyngeal muscles and sensory nerves. Hemostasis is typically achieved using coblation, microdebrider, or low-thermal electrosurgery.
∘ Pain Implications: Significantly lower postoperative pain reduced analgesic use, and faster resumption of oral intake and normal activities in children compared to extracapsular techniques.
∘ Pain Management Considerations: NSAIDs and acetaminophen are generally sufficient; opioids are rarely required [73,74].

#### 5.1.3. Electrocautery/Bovie Tonsillectomy

∘ Description: Uses high-frequency electric current for cutting and coagulation. Offers excellent intraoperative hemostasis but may cause thermal tissue injury.
∘ Pain Implications: Associated with increased thermal damage and higher postoperative pain compared to low-temperature techniques such as coblation.
∘ Pain Management Considerations: Multimodal analgesia (acetaminophen + NSAIDs ± opioids) is commonly required [72].

#### 5.1.4. Coblation (Cold Ablation) Tonsillectomy

∘ Description: Low-temperature radiofrequency energy removes tonsillar tissue while minimizing damage to surrounding muscle and nerves. Hemostasis is achieved with low thermal coagulation.
∘ Pain Implications: Children undergoing coblation tonsillectomy discontinue prescription opioids one day earlier than those undergoing electrocautery and use approximately half as many opioid doses. Parents report the postoperative experience as “better than expected.” [71,74].
∘ Pain Management Considerations: NSAIDs and acetaminophen are generally sufficient; opioids rarely required.

#### 5.1.5. Harmonic Scalpel Tonsillectomy

∘ Description: Uses ultrasonic energy for dissection and coagulation, reducing thermal spread.
∘ Pain Implications: Moderate postoperative pain; less thermal injury than electrocautery.
∘ Pain Management Considerations: NSAIDs and acetaminophen are usually adequate [72].

#### 5.1.6. Microdebrider Tonsillectomy

∘ Description: Partial removal of tonsillar tissue, often in an intracapsular approach.
∘ Pain Implications: Associated with reduced pain and faster recovery compared to traditional techniques.
∘ Pain Management Considerations: NSAIDs and acetaminophen suffice in most cases [74].

### 5.2. Adenoidectomy Techniques

#### 5.2.1. Cold Curettage Adenoidectomy

∘ Description: Adenoid tissue is removed with a curette or suction curette.
∘ Pain Implications: Mild postoperative pain; generally well-tolerated.
∘ Pain Management Considerations: NSAIDs or acetaminophen are sufficient [72].

#### 5.2.2. Electrocautery/Radiofrequency Adenoidectomy

∘ Description: Adenoid tissue is ablated with electrocautery or low-temperature radiofrequency energy.
∘ Pain Implications: Slightly more pain than cold curettage, but still mild.
∘ Pain Management Considerations: Scheduled acetaminophen or NSAIDs; pre-emptive analgesia may be beneficial [71].

Key Points

- Intracapsular tonsillectomy offers the most significant reduction in postoperative pain and opioid use in children.
- Coblation provides significant benefits over traditional electrosurgery in terms of opioid use and patient/parent satisfaction.
- Choice of surgical technique and hemostasis method directly affects postoperative pain, analgesic requirements, and recovery speed.

Future Directions

The evolution of pain management strategies for pediatric tonsillectomy is poised to focus on refining multimodal, opioid-sparing protocols through technological integration, precision medicine, and the validation of novel techniques. The established efficacy of Enhanced Recovery After Surgery (ERAS) principles, which combine scheduled acetaminophen, NSAIDs, and regional anesthesia, provides a robust foundation for this evolution [2,6]. Future efforts will likely concentrate on optimizing each component of this approach.

A primary direction involves the standardization and widespread adoption of advanced regional techniques, particularly with technological assistance to improve efficacy and safety. The recent demonstration that ultrasound-guided glossopharyngeal nerve blocks provide superior and more reliable analgesia than landmark-based techniques underscores a significant opportunity [70]. Future research should focus on developing standardized protocols for ultrasound-guided blocks, training modules for surgeons and anesthesiologists, and investigating the synergistic effects of combining different nerve blocks (e.g., maxillary and glossopharyngeal) for more comprehensive pain control.

The future of pain management in pediatric tonsillectomy is poised to move beyond standardized protocols toward a paradigm of personalized, opioid-free precision medicine, leveraging advancements in pharmacogenomics and immersive technology. The established foundation of multimodal analgesia, which effectively combines acetaminophen, NSAIDs, and regional techniques, will be enhanced by tailoring interventions to the individual patient’s genetic profile and psychological state.

A critical direction involves the clinical implementation of preemptive pharmacogenetic testing to guide analgesic selection and dosing. As demonstrated by the clinical application of CYP2D6 genotyping to avoid codeine in poor and ultrarapid metabolizers, there is a clear precedent for using genetic data to prevent adverse drug reactions and improve efficacy [58,59]. Future protocols will likely expand this concept to create comprehensive genetic profiles that inform the use of other opioids and non-opioid adjuvants, ensuring the right drug at the correct dose for the right patient and fundamentally eliminating genetically mediated toxicity.

Concurrently, the integration of non-pharmacologic, digital therapeutics will become a standard component of perioperative care. Evidence from meta-analyses confirms that immersive virtual reality (VR) is an effective tool for reducing procedural pain and anxiety in children by engaging multiple senses and demanding cognitive attention, thus acting as a powerful distractor [75]. Future research should focus on implementing VR not just for needle procedures but also for managing postoperative pain at home, providing a potent, zero-risk analgesic adjunct that empowers children and reduces reliance on medications.

Laser tonsillectomy is a promising surgical approach to reduce postoperative pain and hasten recovery. Several randomized and comparative studies report lower early postoperative pain scores, reduced intraoperative blood loss, and faster return to normal diet with laser techniques compared with conventional dissection or cautery in children and adults. Systematic reviews and comparative analyses suggest laser and other low-thermal techniques may offer analgesic advantages, though results vary by laser type and study design. Larger, contemporary randomized trials are needed to confirm these benefits and to evaluate hemorrhage risk and long-term outcomes before routine adoption [76,77,78,79,80]. Quality improvement and implementation science are also transforming perioperative care. A 2025 study described the development and implementation of a perioperative pediatric tonsillectomy pain management toolkit designed to standardize multimodal analgesia, enhance recovery pathways, and improve caregiver education across institutions. Such toolkits may facilitate consistent opioid-sparing practice, reduce variability in pain control, and promote adherence to evidence-based protocols in pediatric tonsillectomy [81].

Ultimately, the goal is a fully integrated and personalized recovery pathway. The future standard of care will likely involve a pre-operative genetic workup to create a tailored analgesic plan, combined with prescribed digital VR therapy for home use. This approach promises to maximize safety and efficacy, minimize opioid exposure to zero for most patients, and transform the tonsillectomy recovery experience for children and their families.

## Figures and Tables

**Table 2:** Structured Home Pain Management and Supportive Measures.

Component	Recommended Practice	Supporting Evidence/Guidelines
Scheduled dosing	Alternate acetaminophen and ibuprofen for the first 48 h	AAO-HNS 2019 [1] PROSPECT 2021 [30]
Rescue analgesia	Short opioid (e.g., oxycodone) only if uncontrolled pain; avoid codeine/tramadol	FDA safety warnings; AAO-HNS 2019 [1]
Hydration & diet	Encourage frequent fluids; soft diet	Reduces dehydration risk, improves recovery [1]
Caregiver education	Written instructions with dosing charts, warning signs	Improves adherence and outcomes [2]
Non-pharmacologic adjuncts	Distraction, music therapy, relaxation techniques	Low-risk, caregiver-implementable [2]
Follow-up/safety	24-h access to provider; monitor for secondary hemorrhage (days 5–10)	Guideline consensus) [1,33]

## Data Availability

Not applicable.
